# Playful learning, inclusion, and belonging: a perspective on engaged classrooms

**DOI:** 10.3389/frcha.2026.1736138

**Published:** 2026-04-13

**Authors:** Stacia Faith Jackson, Bo Stjerne Thomsen

**Affiliations:** 1 LEGO Education, Billund, Denmark; 2 MIT Media Lab, Massachusetts Institute of Technology, Cambridge, MA, United States

**Keywords:** belonging, children, curriculum development (education), education, inclusion, learning desgin, playful learning, universal design for learning (UDL)

## Abstract

This perspective argues that supporting child and adolescent mental health in schools requires classrooms that foster belonging, engagement, and agency for every learner. Upon reading this article, we propose that the research field explores more rigorously into the connections between classroom engagement, playful learning, inclusion, and sense of belonging. In this perspective, the concept of belonging is central to the idea of mental health. Our viewpoint is that playful learning is a promising pathway to support students who report feeling excluded or unsupported in their classroom. While perhaps not a cure-all for all students in all classrooms, it is an area that rightfully deserves more attention as its impact can go beyond the classroom wall. We propose further exploration of the intersection between classroom engagement, playful learning, inclusion, and sense of belonging specifically for primary and secondary school students. By grounding learning in joyful, hands-on, and collaborative experiences, playful approaches can offer a practical means of increasing belonging within the classroom. Drawing on classroom practice and product development, including examples such as LEGO Education Science, we explore how playful learning can support the core learning domains of education into safe and supportive environments that nurture both cognitive and emotional growth. Much like the topic of science itself, the intersectionality between inclusion, learning through play, and belonging is still evolving. In the following pages, we'll explore opportunities for educators, researchers, and policymakers to build systems that reduce teacher burden while strengthening inclusion and engagement. We point to the need for accessible practices and ready-to-use resources that allow teachers to implement playful learning without additional workload. Finally, we outline directions for research and collaboration to further understand and scale approaches that simultaneously advance learning, inclusion, and belonging.

## Introduction

Classrooms across the world are more diverse than ever. They bring together learners with different cultural backgrounds, linguistic capabilities, prior knowledge, and neurodivergent profiles. Yet many schools still rely on one-size-fits-all approaches: uniform pacing, standardized instruction, and expectations that all learners engage in the same way. This rigidity contributes not only to academic disengagement but also to heightened stress and feelings of exclusion. Students may internalize feelings of inadequacy, perceiving themselves as separate from the broader classroom and community context, rather than experiencing a sense of belonging. It is our view that belonging is defined as part of students’ experiencing a sense of being accepted, valued, and able to participate meaningfully in the classroom and is an important subset of their broader mental health and well-being.

Today's classrooms are markedly diverse: in the United States, about 49.6 million students attend public schools (1), and roughly 7.5 million (15%) receive additional supports and services (2). Research shows wide variation in motivation and modes of engagement (3, 4), and learners with disabilities or additional needs must be intentionally included in the learning experience (5, 6). To include all students and provide appropriate supports, it is important to identify specific needs and challenges, which requires naming common needs—e.g., specific learning disabilities, speech or language impairments, health impairments, autism spectrum disorder, developmental delay, intellectual disability, emotional disturbance, and hearing impairment (7)—and aligning supports accordingly. “Specific learning disability” is defined as “a disorder in one or more of the basic psychological processes involved in understanding or using spoken or written language that may manifest itself in an imperfect ability to listen, think, speak, read, write, spell, or do mathematical calculations” (8); examples include dyslexia (reading), dysgraphia (impairment in written expression), and dyscalculia (mathematics) (9).

Teachers find themselves at the center of this dilemma. They are expected to differentiate for increasingly diverse learners while facing constraints of time, accountability pressures, and limited resources. This load is unsustainable and risks driving burnout while leaving the underlying structural issues unaddressed.

What is needed are pedagogical pathways that simultaneously support student engagement, belonging, and well-being, while being feasible for teachers to implement.

Playful learning has emerged as a promising pathway. Defined by joy, meaning, active engagement, iteration, and social interaction (10), playful learning offers more than enrichment: it provides a structured approach that integrates learning, inclusion, and psychosocial support. Playful learning, while part of the play spectrum, is a separate category of play. This is a category reserved for the education space and is often embedded in various pedagogical approaches, including active learning, inquiry-based learning, problem-based learning, project-based learning and the list goes on.

### The case for inclusive and engaging classrooms

This underscores the necessity of comprehending engagement within the classroom context. Engagement, which functions as a mechanism influencing both learning and a sense of belonging, also serves as an observable indicator of classroom activities. It can be analysed through the dimensions of physical, cognitive, and affective involvement. This multi-level unengagement is a robust predictor of positive academic outcomes (11–14), while disengagement is linked to disaffection, dropout, and academic failure (15). As students mature, the ties between classroom engagement and mental health become more pronounced (16). Engagement is also greater belonging and productivity (15).

A critical dimension of engagement is ensuring that learning environments are inclusive of all students. Because students cannot engage if they are excluded, inclusion is a precondition for engagement (17).

Taken together, these realities indicate a necessary shift away from one-size-fits-all methodologies and toward intentionally inclusive practice that also bolster students’ mental health (18–20). Inclusion helps ensure that all learners receive the supports they need (21). In practical terms, teachers are expected to differentiate—to “ensure all learners have the support they need” (22). Differentiation entails proactive adjustments to curricula, methods, resources, activities, and products to meet diverse needs (23), with materials and approaches evolving as cohorts change; this can mean revisiting goals, content, pace, progression, and working methods over time (24).

This task can be overwhelming for teachers. Given persistent challenges in teacher retention, there is an opportunity to alleviate some of teachers’ physical and mental workload (25–28). Even so, students must feel supported: nearly half report that clear explanations would have a major impact on their motivation in math, science, engineering, and technology courses (22). When teachers meet students where they are, they foster high-quality relationships characterized by closeness and low conflict, which positively affects academic motivation (29–32).

### Playful learning as a potential pathway

Classrooms that strive for inclusion and academic rigor need designs that invite every learner to participate, sustain motivation, and feel safe enough to take intellectual risks. Addressing these intertwined challenges requires pedagogical models that make engagement and inclusion sustainable for teachers while advancing motivation and mental health for students. Playful learning, embedded in everyday lessons, offers a promising model—fostering creativity and engagement while meeting diverse needs within the same classroom.

When lessons are intentionally planned so that curiosity, exploration, social interaction, and iteration are designed for, students are more likely to engage, experience belonging, and build the self-regulatory skills that protect mental health—without lowering expectations for learning (10, 33). A long tradition of evidence shows that play is a context for coping and self-regulation (34–38). Meta-analyses of play therapy report reliable benefits for children's internalizing and externalizing difficulties and improved adjustment and relationships (39, 40). In everyday classrooms, this translates into structured opportunities to explore, test, and revise—especially with hands-on materials—so students can externalize thinking, regain a sense of control, and re-engage after challenges.

Two design frameworks help teachers operationalize this approach. First, the play spectrum situates pedagogy on a continuum—from explicit instruction through guided play to free play—so teachers can move fluidly among modes as concepts, learner needs, and emotional demands shift (33). Second, the framework from Learning through Play specifies five mutually reinforcing features—joy, meaning, active engagement, iteration, and social interaction—that teachers can plan for in each lesson, creating predictable, inclusive cycles of exploration and reflection that help regulate uncertainty and sustain attention (10). These sit comfortably alongside inquiry models such as the 5E framework and with autonomy-supportive instruction, both linked to higher motivation and achievement (41–43).

Agency is pivotal for inclusion and belonging. When students experience consequential but bounded choices—how to approach a task, which representation to use, when to iterate—they develop competence and ownership. Classic theory and meta-analytic evidence show that structured choice increases intrinsic motivation and performance, especially when choices are meaningful and aligned to clear goals (3, 44). Hands-on materials (manipulatives, construction kits, lab equipment) are powerful in this regard: meta-analytic and synthesis work in science education shows that concrete, well-scaffolded activity improves conceptual understanding when it is instructionally meaningful and embedded in reflection rather than decorative add-ons (45–47). In practice, it means meeting students where they are in their learning journey and teachers respecting students’ current knowledge—often summarized as “essential for some, beneficial for all.”

### Limitations on work

Facilitating playful learning isn't a one-size-fits-all solution, nor will it quickly transform classroom culture or educational norms. Implementing guided play, which requires balancing student agency with structured guidance, presents significant challenges for teachers. It is crucial not to underestimate the complexity of this undertaking. Changing school culture involves navigating entrenched practices and beliefs, making it akin to the broader difficulties faced in change management across various fields. Unfortunately, research on how to effectively manage these shifts in pedagogy is limited, highlighting a need for the academic community to explore these areas more comprehensively.

On the other side of this, we know that playful learning anecdotally engages students. The anecdotal stories of students feeling more motivated to learn, not realizing they were learning while in an activity, and not wanting to stop when time was up abound. However, ample opportunities still exist for more quantitative research or longitudinal studies to deepen and extend this field.

### Illustrative example from science education

With this information in mind, we’d like to offer an example of inclusive, playful learning experiences that offer a sense of belonging to students. In looking to find similar products, it is difficult to find learning experiences that have inclusion and differentiation included from the start of the design experience. It is necessary to look for products and learning experiences that are designed for the general education classrooms and include the inclusion and differentiation from the beginning of the product development. This is in direct contrast to several differentiation and accessibility options that make an existing learning experience accessible.

The design of LEGO® Education Science illustrates how playful learning, inclusion, and the integration of Universal Design for Learning can be woven into structured classroom practice. By embracing the UDL framework, educators can design lessons that not only meet content-specific goals but also foster engagement, collaboration, and creativity among all students. The structured yet flexible approach of the 5E Instructional Model, combined with hands-on materials and the inquiry-driven nature of LEGO Education Science, empowers learners to explore scientific concepts actively and meaningfully.

- Visual Supports: Tactile minifigures aid visually impaired users, aligning with UDL by offering multiple engagement methods and enhancing accessibility.
- Progress Tracking: A progress bar in lessons helps students, especially those with autism, understand their learning journey, reducing anxiety and providing structure.
- Consistent Structure: The 5E instructional model creates predictability, aiding students who struggle with transitions and allowing them to focus better.
- Segmented Learning: Lessons are broken down to accommodate varying processing speeds, with extended segments for collaborative activities.
- Multi-Modal Instruction: Content is delivered through visual, written, and oral methods, supporting diverse language development and enhancing engagement.
- Vocabulary in Context: New terms are introduced with definitions, aiding students with dyslexia and English language learners in comprehension.
- Flexible Assessment: Adaptable lesson plans encourage a growth mindset, allowing students to learn from failures.
- Real-World Relevance: Connections to real-world applications help underrepresented students see themselves in STEM fields, increasing motivation.
- Physicality of LEGO: The hands-on experience builds fine motor skills and allows for varied expression through manipulation of LEGO bricks.
- Intentionality in Teaching: Teachers have the flexibility to adapt lessons, fostering an inclusive environment for all learners.

### Not a single solution but a practice to incorporate into teaching

The world of education does not stop and wait for research to catch up with practice. The class bell doesn't wait for new practices to be disseminated. While research continues to evolve, it is necessary to look to concrete strategies and tactics teachers can use in the classroom to propel inclusion and engagement in the classroom. An often-repeated chorus is that teachers know their students the best and know what those students need to learn. Teachers will spend approximately 1,200 h with their students each year (48). This is an incredible amount of time that teachers have to get to know their students, including the best way each student learns.

Individual teachers can spur change within their classrooms, which is powerful and transformative. Teachers can differentiate and support on a student-by-student basis but as we've noted, the cognitive and emotional load becomes heavy. How do we move toward an educational landscape that is high-quality, enables every student to succeed, and does not add to teachers' work? Perhaps the first area to examine is the teacher's role itself. As mentioned above, the workload of teachers is immense, and the cognitive weight of that should not be underestimated. Teachers face competing priorities that extend far beyond the confines of the classroom.

The materials that are produced for the classroom must be created in a way that supports students holistically. This means embedding the Universal Design for Learning guidelines from the beginning and not attempting to retroactively align. The ready-to-use inclusive materials must include multiple means of engagement, representation, and action and expression of both the content and the responses to the content. For teachers to be able to support students, then the tools they are equipped with must be ready-to-go right out of the box. By using such features as visual scaffolds, multimodal interactions, and varied forms of expression, teachers can focus on their students rather than redesigning their materials.

The responsibility of differentiation and adaptation should not rest solely on the shoulders of teachers. Instead, it is imperative that those involved in curriculum and content development take on this challenge. To truly enhance the learning experience, we must amplify diverse voices in the development process. There is a wealth of expertise and experience globally, particularly from the disability community, which can significantly inform our practices. The phrase “nothing about us without us” resonates deeply here, emphasizing that individuals with disabilities must be actively involved in decisions that affect their education. This principle is echoed in the Convention on the Rights of Persons with Disabilities (CRPD), which advocates full participation and inclusion in society.

Related to this, it is important for teachers to engage in high-quality professional learning experiences that go beyond passive information reception. Instead, teachers should experience the same high-quality, multi-modal learning opportunities, like those that are most effective for their students.

It is time for curriculum developers to produce and disseminate materials and learning experiences that do not require additional scaffolding, augmentation, or adjustments. Creating curriculum that includes options for differentiation and inclusion does not adequately meet the need. This requires looking towards the future to agitate.

## Discussion: future directions

Perhaps the most pressing future work within this area is the need to build upon the existing research and develop a more substantial link between engagement, inclusive classrooms, and belonging. While this perspective has begun the task of linking the three topics together, bridging the gaps with playful learning, more, explicit research, both desk research and field study, is needed. It should also be noted that this research should not be isolated to early childhood education, a field filled with research on play. Instead, it is imperative to look at inclusion, engagement, and belonging from a variety of viewpoints, including students (elementary, middle, and high school), teachers and educators, and parents. This multi-faceted approach will allow the most comprehensive overview and deepen the understanding of this intersection.

To deepen understanding in this area, the principle “nothing about us without us” is crucial. It's essential to ensure that all students’ voices are actively heard, including those who are neurotypical and those with disabilities. Equity must be integrated from the outset, emphasizing co-design with disability communities. Engaging with student disability communities allows their experiences and insights to shape the learning environment.

Supporting students effectively requires interdisciplinary collaboration across fields like education, psychology, and pediatric and adolescent mental health. This collaboration should include classroom ethnography, physiological stress measures, and student co-creation.

Evidence suggests that inclusive pedagogy and a sense of belonging are mutually beneficial and reinforcing in classrooms. However, more concrete connections and examples are needed to advance this field of research. Playful learning fosters a protective climate by providing routine opportunities for social interaction, meaningful choices, and low-stakes iteration, all while maintaining high expectations. This includes the discussion and grappling with of two key questions:

1. What are the key observable behaviors and characteristics that define inclusive, playful learning in real classrooms and how do they vary across context?
2. If a student is meaningfully engaged in the classroom, what does that mean in terms of their mental health, self-image, and desire to attend school?

Real change requires a dual pathway. At classroom level, predictable inquiry routines (e.g., 5E), multimodal access, and small, consequential choices can normalize participation and reduce performance anxiety. At system level, procurement criteria that require UDL by design, professional learning that models playful pedagogy, and accountability that attends to belonging and classroom climate—alongside academics—can make these practices sustainable rather than additive.

Taken together, playful learning is not an enrichment sidebar but a coherent approach that integrates engagement, inclusion, and well-being. Embedding playful pedagogy through UDL and equipping teachers with ready-to-use materials and predictable routines offers a feasible route to classrooms where learners feel they belong, can take intellectual risks, and are more likely to thrive academically and emotionally.

## Data Availability

The original contributions presented in the study are included in the article/Supplementary Material, further inquiries can be directed to the corresponding author.
